# Supplementary figures and images for: Focused Ultrasound Stimulates ER Localized Mechanosensitive PANNEXIN-1 to Mediate Intracellular Calcium Release in Invasive Cancer Cells
Source: Front Cell Dev Biol. 2020 Jun 23;8:504. doi: 10.3389/fcell.2020.00504 (PMC7325310; doi:10.3389/fcell.2020.00504)

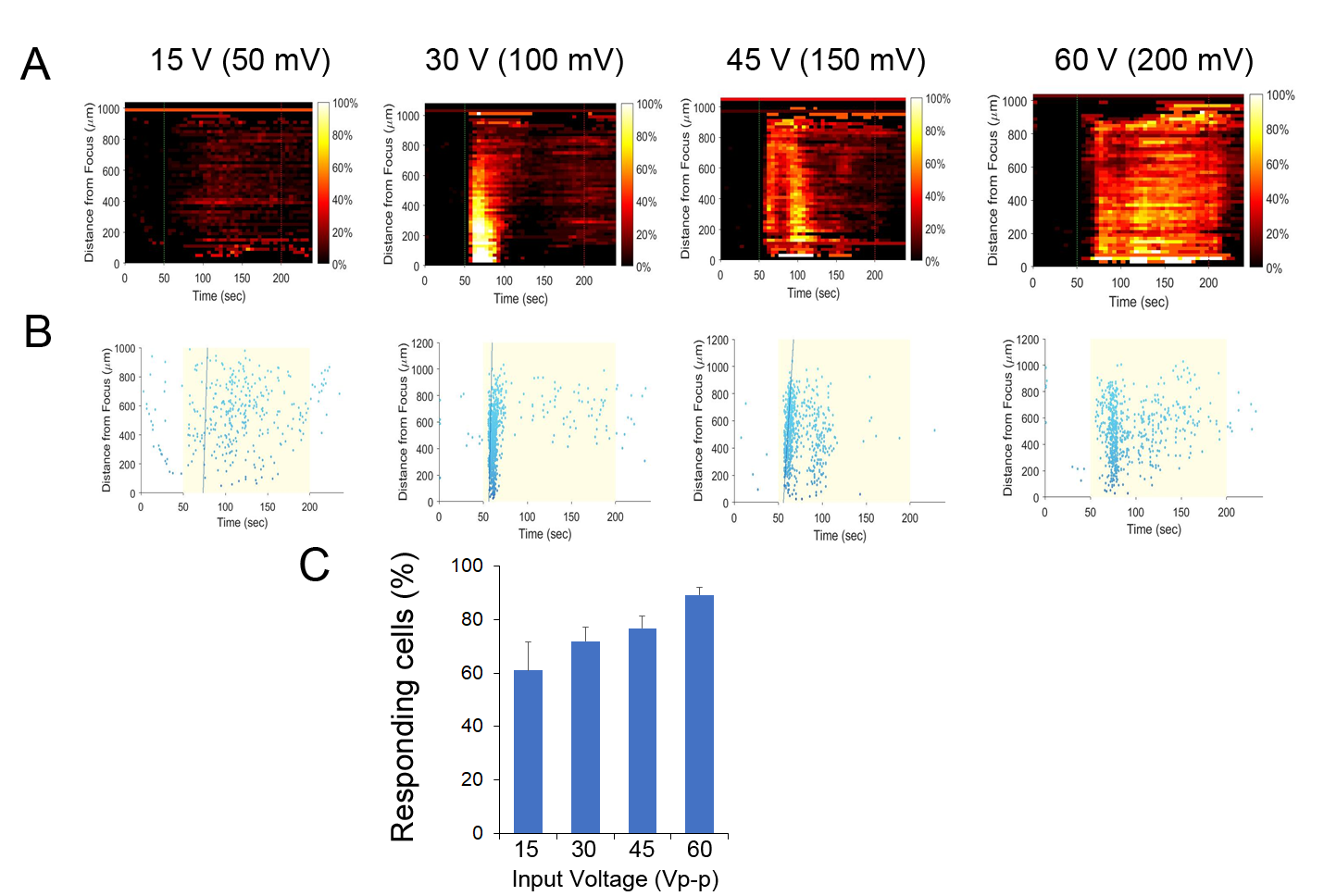

Supplement: FIGURE S1 — Effect of FUS stimulation amplitude on PC-3 cell calcium response using 3-MHz transducer. All stated voltages represent peak-to-peak amplitude (Vp-p). Values in parentheses indicate the mV at each voltage, as measured by a hydrophone. (A) 2-D histograms showing the percentage of responding cells over time. (B) Scatter plots showing the time at which each cell first responded to the stimulus (each dot represents a responding cell). (C) Quantitative percentage of responding cells. n = 3. Error bars, s.e.m., n represents biological replicates. [file Image_1.TIF]

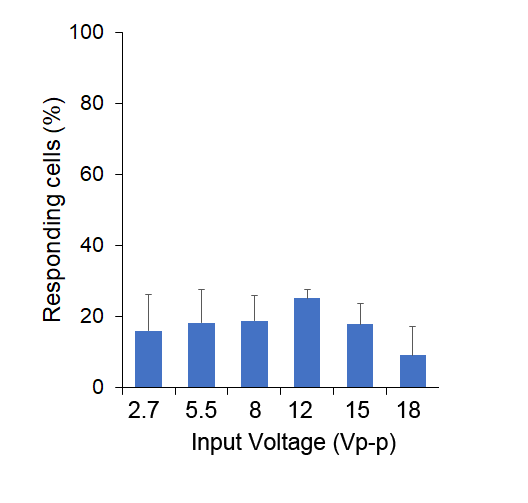

Supplement: FIGURE S2 — Effect of FUS stimulation amplitude on HEK cell calcium response using 46-MHz transducer. All stated voltages represent peak-to-peak amplitude (Vp-p). Values in parentheses indicate the mV at each voltage, as measured by a hydrophone. Quantitative percentage of responding cells. n = 3. Error bars, s.e.m., n represents biological replicates. Some spontaneous response background was occasionally shown, so the percentages of responding cells are ∼10%. However, the calcium response in HEK cells was not altered by different FUS stimulation amplitude, which is different from PC-3 cells. [file Image_2.TIF]

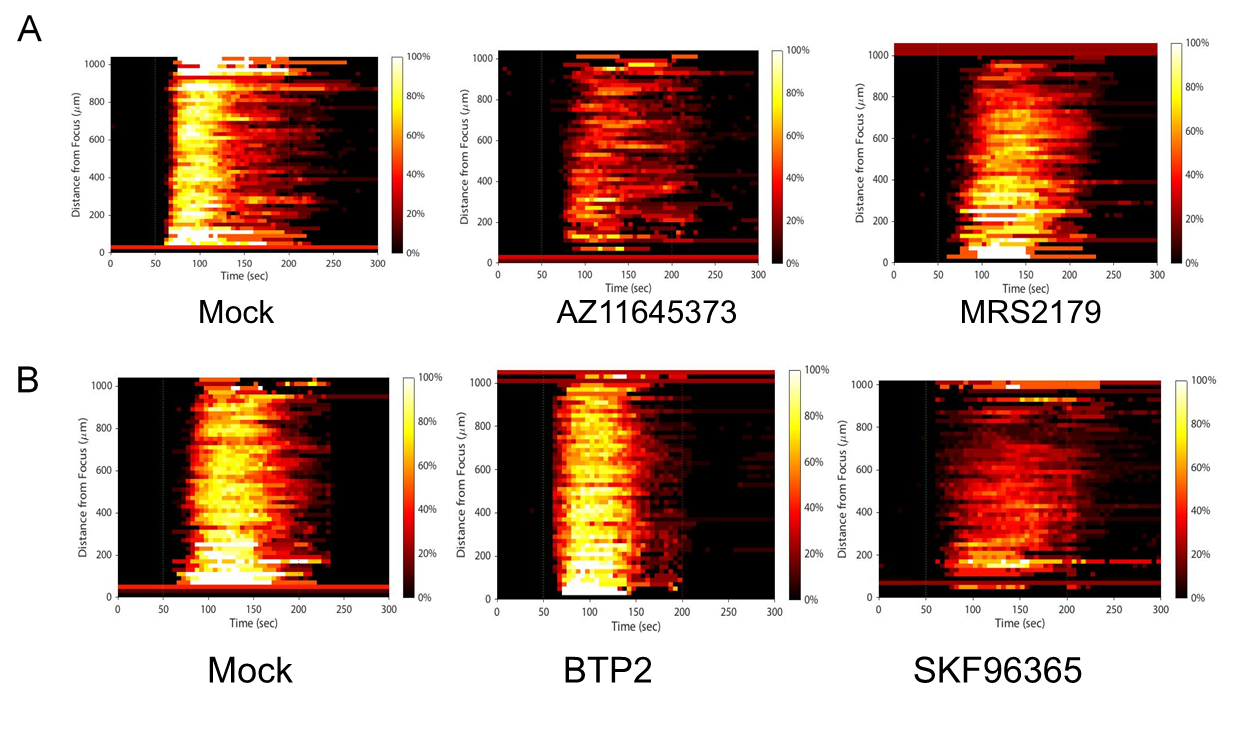

Supplement: FIGURE S3 — Effect of treatment of inhibitors on PC-3 cell calcium response. 2-D histograms showing the percentage of responding cells over time. (A) Effect of treatments of P2 receptor inhibitors on PC-3 cell calcium response. (B) Effect of treatments of Ca2+ influx inhibitors on PC-3 cell calcium response. These did not change the calcium response. [file Image_3.TIF]

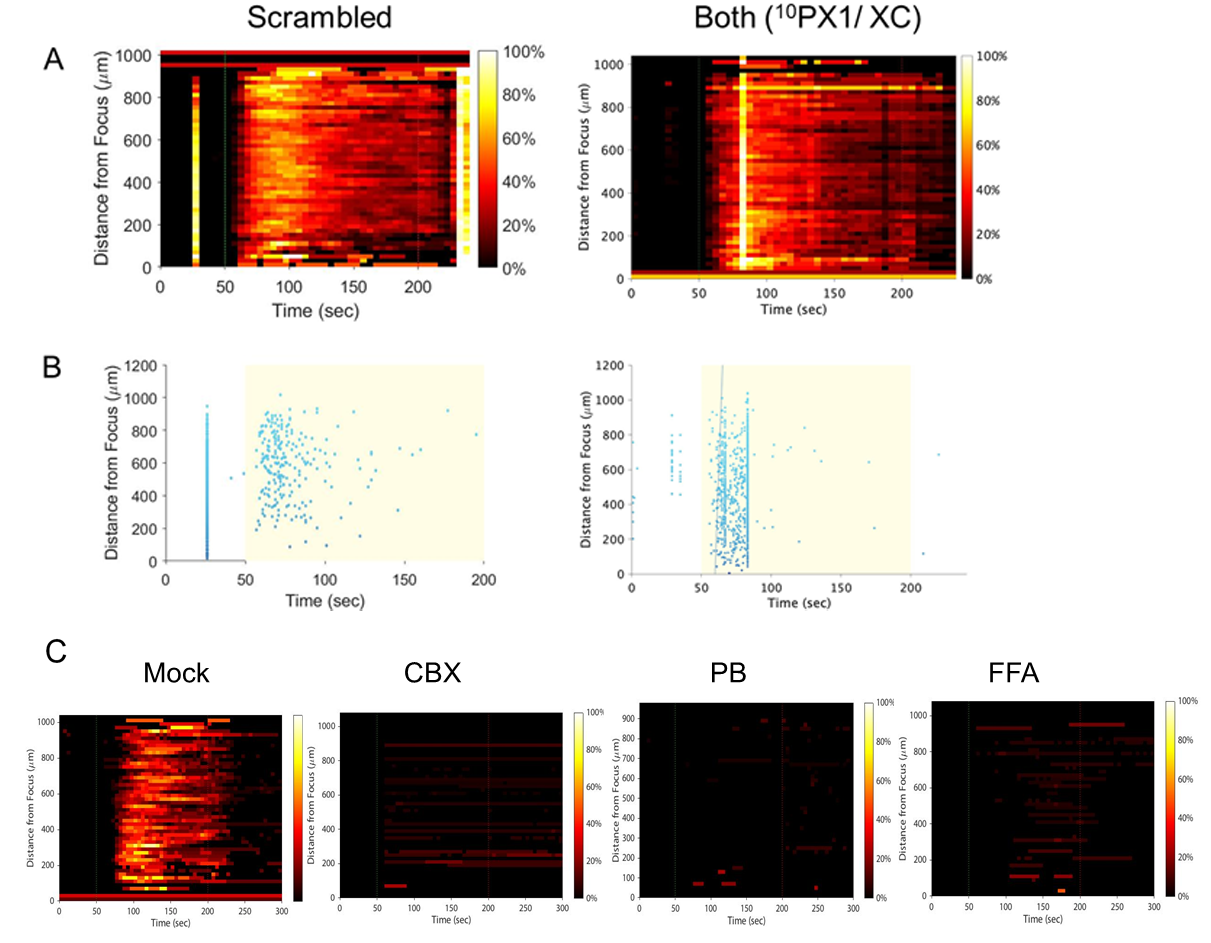

Supplement: FIGURE S4 — Effect of both treatment of 10PX1 and XC on PC-3 cell calcium response using 46-MHz transducer. (A) 2-D histograms showing the percentage of responding cells over time. (B) Scatter plots showing the time at which each cell first responded to the stimulus (each dot represents a responding cell). (C) Effect of treatments of CBX, PB and FFA. The histograms showed the percentage of responding cells over time. Treatment of CBX, PB or FFA in PC-3 cells abolished Ca2+ responses. [file Image_4.TIF]

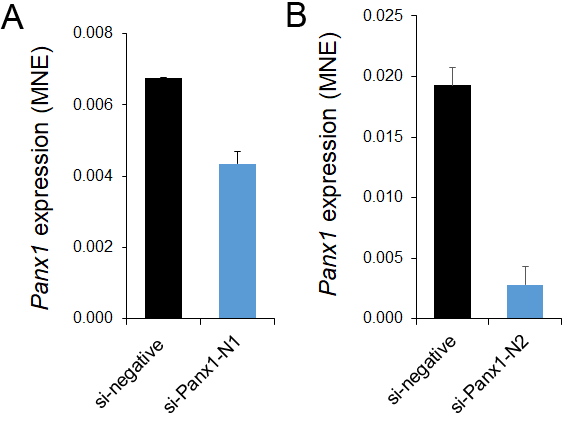

Supplement: FIGURE S5 — Quantitative RT-PCR analysis of WT PANX1 transcript expression in PC3 cells transfected two independent siRNAs that specifically target FL PANX1, as described previously; n = 2. Error bars, s.e.m., si-PANX1-N1 (L-018253-00) showed <35% reduction (A) so we did not use it. Another si-PANX1-N2 (D-018253-02) was used in most experiments and called as ‘si-PANX1’ (B). The variations of reduction occurred because of cell heterogeneity. [file Image_5.TIF]
