# Supplementary material for: Focused Ultrasound Stimulates ER Localized Mechanosensitive PANNEXIN-1 to Mediate Intracellular Calcium Release in Invasive Cancer Cells
Source: Front Cell Dev Biol. 2020 Jun 23;8:504. doi: 10.3389/fcell.2020.00504 (PMC7325310; doi:10.3389/fcell.2020.00504)
Supplement: TABLE S1 — Pharmacological agents used to investigate the mechanism of FUS-induced calcium rise in PC-3 cells. [file Table_1.pdf]

Table S 1. Pharmacological agents used to investigate the mechanism of FUS-induced calcium rise in PC-3 cells.

Blocker/Inhibitor	Company	Effect	Concentration (μM)	Loading Time (min)
Suramin	Sigma-Aldrich	P2X and P2Y receptor antagonist	100	15
Apyrase (grade I)	Sigma-Aldrich	Degradation of extracellular ATP	20-50 units/ml	15
Flufenamic acid	Tocris	GAP junction inhibitor	100-200	30
PPADS	Sigma-Aldrich	P2 purinergic receptor antagosist	100	30
Probenecid	Probenecid	Pannexin1 inhibitor	10	15-30
Carbenoxolone (CBX)	Sigma-Aldrich	Pannexin1 inhibitor	200	15-30
^{10}Panx1	Tocris	Pannexin1 inhibitor	100	15-30
Scrambled ^{10}Panx1	Tocris	Control for ^{10}Panx1	100	15-30
Thasigargin	Sigma-Aldrich	Intracellular Ca^{2+} store inhibitor	10	30
BTP2	Sigma-Aldrich	CRAC (STIM1) inhibitor	3	30
Xestospongin C	R&D Biotec.	IP3Rs inhibitor	2	30
SKF96365	SKF96365	TRP inhibitor	30	30
MRS2179	Sigma-Aldrich	P2Y1 receptor antagonist	10	30
AZ11645373	Sigma-Aldrich	P2X7 selective inhibitor	3	20-30
Cytochalasin D	Sigma-Aldrich	actin filament inhibitor	2	30
Nocodazole	Sigma-Aldrich	microtubule inhibitor	1	30
ML-7	Cayman Chem	actomyosin contractility inhibitor	5	30
(-)-Blebbistatin	Cayman Chem	actomyosin contractility inhibitor	5	30
